# Defining post-acute COVID-19 syndrome (PACS) by an epigenetic biosignature in peripheral blood mononuclear cells

**DOI:** 10.1186/s13148-022-01398-1

**Published:** 2022-12-14

**Authors:** Frida Nikesjö, Shumaila Sayyab, Lovisa Karlsson, Eirini Apostolou, Anders Rosén, Kristofer Hedman, Maria Lerm

**Affiliations:** 1 Department of Respiratory Medicine in Linköping, Department of Biomedical and Clinical Sciences, Linköping University, Linköping, Sweden; 2 Division of Inflammation and Infection, Department of Biomedical and Clinical Sciences, Faculty of Medicine and Health Sciences, Linköping University, Campus US, Medical Microbiology, Lab1, Floor 12, 581 85 Linköping, Sweden; 3 Division of Cell Biology, Department of Biomedical and Clinical Sciences, Linköping University, Linköping, Sweden; 4 Department of Clinical Physiology, Department of Health, Medicine and Caring Sciences, Linköping University, Linköping, Sweden

**Keywords:** Post-acute COVID-19 syndrome, PACS, COVID-19, SARS-CoV-2, PBMC, Epigenetics, DNA methylation, Biosignature

## Abstract

Post-acute COVID-19 syndrome (PACS) has been defined as symptoms persisting after clearance of a COVID-19 infection. We have previously demonstrated that alterations in DNA methylation (DNAm) status persist in individuals who recovered from a COVID-19 infection, but it is currently unknown if PACS is associated with epigenetic changes. We compared DNAm patterns in patients with PACS with those in controls and in healthy COVID-19 convalescents and found a unique DNAm signature in PACS patients. This signature unravelled modified pathways that regulate angiotensin II and muscarinic receptor signalling and protein–protein interaction networks that have bearings on vesicle formation and mitochondrial function.

**Supplementary Information:**

The online version contains supplementary material available at 10.1186/s13148-022-01398-1.

## Introduction

In some individuals, the acute phase of COVID-19 causes lethal disease, while others may experience only mild symptoms or remain asymptomatic. Also, in those with a mild course of infection, complications may persist after clearance of infection. Symptoms continuing more than 4 weeks after the infection have been defined as long-term complications of COVID-19, while symptoms persisting beyond more than 12 weeks are often referred to as “Post-acute COVID-19 Syndrome” (PACS) [1]. Commonly reported PACS symptoms include, but are not limited to, physiological (muscle weakness, exercise intolerance, postural orthostatic tachycardia syndrome, palpitations, dyspnoea, dry cough, chest pain, loss of smell and taste), psychiatric (insomnia, anxiety, depression, nightmares) and/or cognitive dysfunction (fatigue, memory problems, light sensitivity, sleep disturbances) [1]. The discussion about symptom subgroups in PACS is ongoing, where grouping based on affected organ(s) has been proposed. Symptoms can fluctuate, persist or fade over time. A correlation between disease severity during the acute infection and PACS has been reported [1], though not limited to severe infection. Currently, the pathophysiological mechanisms underlying PACS remain largely unknown.

Recently, we demonstrated that healthy COVID-19 convalescents carry a distinct DNA methylation (DNAm) pattern in their peripheral blood mononuclear cells (PBMC) without any shifts in PBMC cell population frequencies [2]. The aim of the current study was to further elucidate the DNAm pattern of PACS patients, by comparing DNAm in PBMC from PACS patients, healthy COVID-19 convalescents (CC19) and controls (Con).

## Methods

We recruited 10 study subjects between 27 and 57 years of age referred to the Department of Clinical Physiology at Linköping University Hospital due to persistent PACS symptoms for more than 12 weeks. The PACS patients had either a previous positive PCR test for SARS-CoV-2 and/or reported symptoms consistent with COVID-19 infection that affected daily life. The PACS group presented with a wide range of symptoms (Additional file 1: Table S1a) and received no hospital care in the acute COVID-19 infection. 7 out of 10 subjects were vaccinated against COVID-19 with Comirnaty (Pfizer/BioNTech) < 4 months prior to inclusion. DNA methylation data from a previous study [2] and serology data from an ongoing study (manuscript in preparation) with subjects who did not report any remaining symptoms after an episode of mild or moderate symptoms of COVID-19 (CC19) along with a matched control group (Con) were used for comparison [2]. CC19s and Cons were collected during spring/summer of 2020. None of the CC19 or Cons (from the previous study) was vaccinated (no vaccines available at that time) (Table S1b) [2]. PBMCs were isolated from 30 ml peripheral blood collected in EDTA tubes and DNA was prepared as described previously [2]. The DNAm analysis was run with the Illumina Infinium Methylation EPIC 850 K BeadChip array (Illumina Inc, San Diego, USA) by the core facility for Bioinformatics and Expression Analysis at Karolinska Institute, Stockholm, Sweden. Bioinformatic analysis was performed (Additional file 1: Supplementary Methods). Suspension multiplex immunoassay (SMIA) analysis for antibodies against CHRM3 in plasma was performed as previously described [2] using human Muscarinic acetylcholine receptor M3 recombinant protein (#MBS955742, My Biosource).

## Results

The data from the PACS patients were compared to the existing data from our previous study [2] on convalescent COVID-19 in which inclusions were done during 2020. The mean PACS symptom duration was 46 weeks (17–66) at the time of inclusion. Statistical comparisons of demographic variables (Additional file 1: Table S1b) revealed no significant differences between the study groups. The PACS subjects all had significant levels of anti-RBD antibodies. PACS samples had significantly stronger IgG responses against SARS-CoV-2 RBD compared to the CC19 group.

**Fig. 1:**
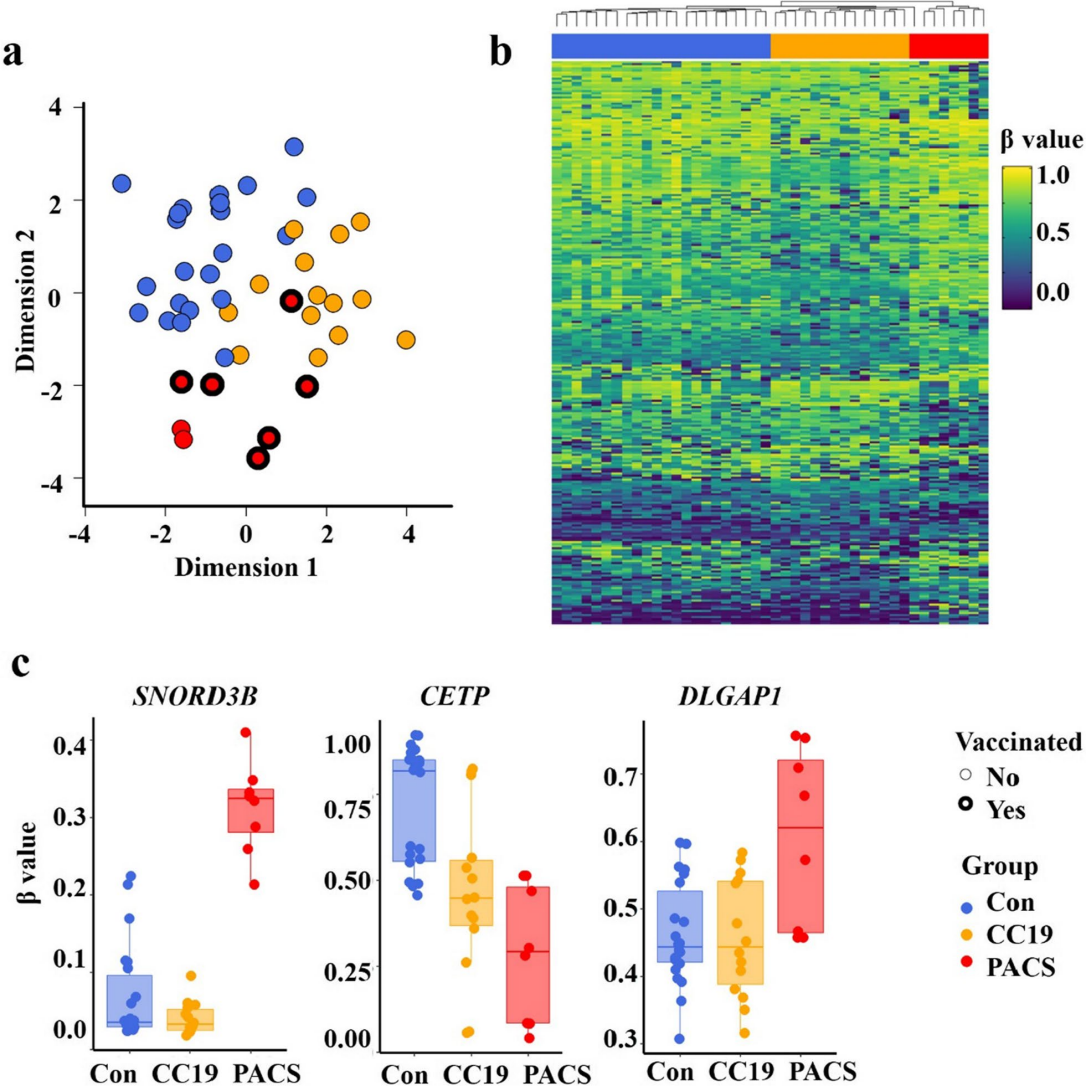
DNA methylation signature analysis of PBMC associated with PACS.** a** Multidimensional scaling (MDS) plot of the three groups; controls (Con, blue), COVID-19 convalescent group (CC19, orange) and patients with post-acute COVID-19 syndrome (PACS, red). Dimension 1 (*x*-axis) and dimension 2 (*y*-axis) show the distances between the samples using the top 1000 most variable CpG positions. Vaccinated (bold lines), non-vaccinated (fine lines). CC19s and Cons were collected during spring/summer of 2020. None of the CC19 or Cons was vaccinated (no vaccines available at that time). **b** Heatmap of differentially methylated CpGs (DMCs) representing an unsupervised hierarchical clustering analysis (Euclidean distance) of individual methylation (*β* values, hypomethylated; blue, hypermethylated, yellow, as indicated) of the 271 identified DMCs. The study groups are coloured as in panel a, and individual samples are arranged as columns. **c** Boxplots of *β* values (*y*-axis) showing the DNA methylation levels at three differentially methylated CpGs mapped to the *SNORD3B*, *CETP*, *DLGAP1* genes, respectively, in each of the study group (*x*-axis)

Of the initial methylation analyses of 865,918 probes, 813,467 probes remained upon filtering with two samples removed due to low quality probes. Multidimensional scaling (MDS) analysis (Fig. 1a) demonstrated that the three groups formed separate clusters, apart from one PACS patient clustering among the CC19 and Con subjects. The cell proportion estimate indicated higher proportion of neutrophils (*t* test* p* value < 0.01) in the PACS group, compared to the other two groups (Additional file 1: Table S2).

**Fig. 2:**
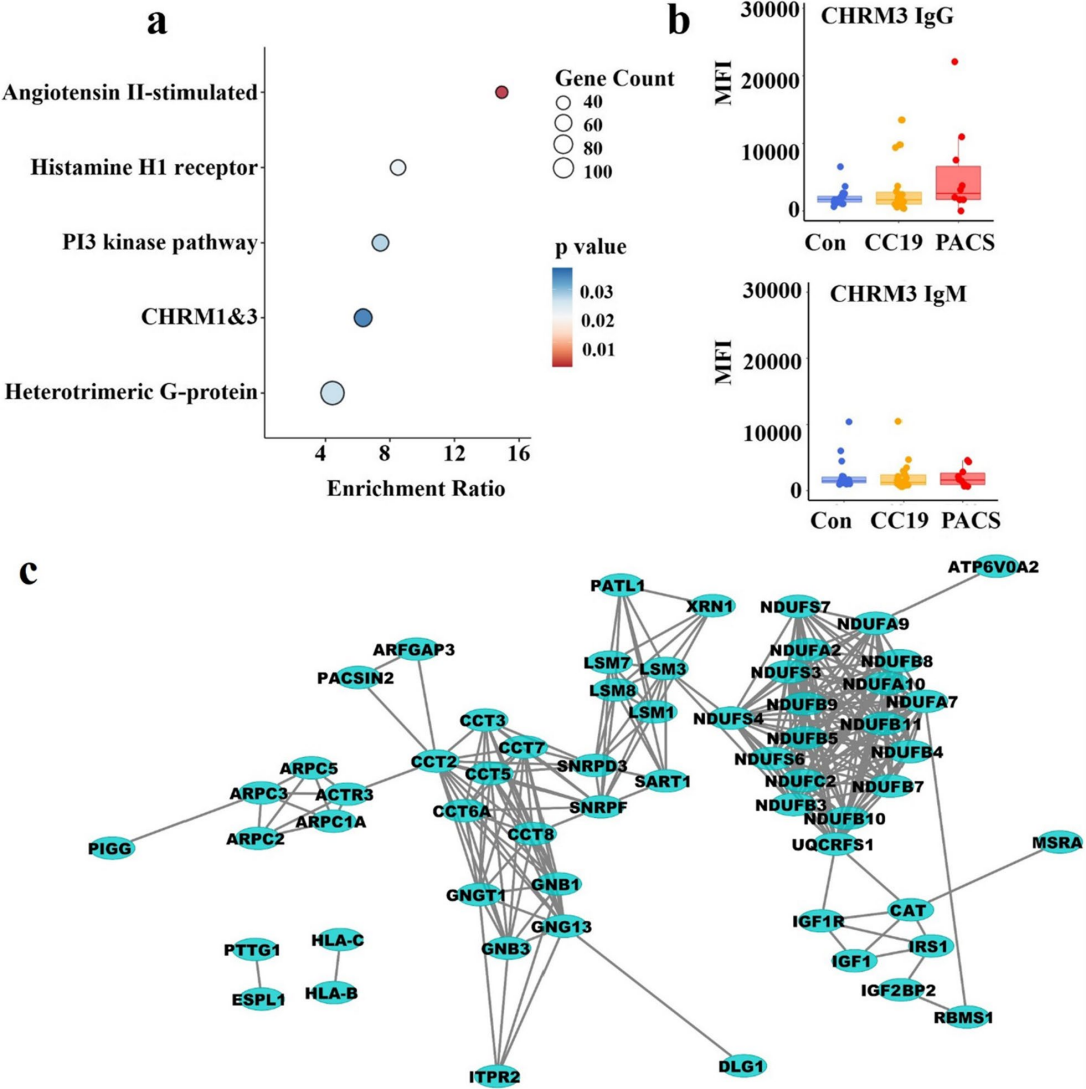
Pathway analysis of PACS and SARS-CoV2 interactome. **a** Dot plot showing the top enriched pathways in PACS resulting from the overrepresentation analysis (Panther, PACS compared to CC19). The gene count is presented as circles, increasing with elevating number of gene count. The significance level is presented as a colour scale from red to blue. Muscarinic acetylcholine receptor M 1&3 antibodies (CHRM1&3). **b** Levels of Muscarinic acetylcholine receptor M3 antibodies (CHRM3) of IgG and IgM class in plasma of each study group, PACS, CC19 and Con. MFI, median fluorescence index. **c** String-DB interactome of PACS DMGs overlapping SARS-CoV2, expanded to fill the gaps until all major modules were connected

We then proceeded to identify differentially methylated CpGs (DMCs) by comparing the PACS group to the CC19 and Con groups, using a threshold of < 0.05 (FDR-adjusted p-value) and a mean methylation difference (MMD) cut-off of > 0.2. (Additional file 1: Fig. S1). We observed 197 (65% hypermethylated) and 98 (57% hypermethylated) DMCs when comparing the PACS individuals’ DNA methylome to the CC19 and Con groups, respectively (Additional file 1: Table S3, Additional file 2: Table S4). We combined the DMCs from the above analysis and generated a heatmap of individual β values of the 271 identified DMCs (Fig. 1b). Each of the DMCs were mapped to their corresponding differentially methylated genes (DMGs) (Additional file 2: Table S4a, b) and the β values of three DMCs selected by significant differences between all groups were mapped to the genes *SNORD3B*, *CETP* and *DLGAP1* are shown in Fig. 1c. Panther pathway enrichment analysis (Fig. 2a) revealed significantly enriched pathways including “Angiotensin II-stimulated signalling through G proteins and β-arrestin”, “Histamine H1 receptor mediated signalling pathway”, “Heterotrimeric G-protein signalling pathway-Gq alpha and Go alpha mediated pathway”, “PI3 kinase pathway” and “Muscarinic acetylcholine receptor 1 and 3 (CHRM1&3) signalling pathway” (Fig. 2a). Since autoantibodies against different G-coupled receptors have been described in PACS [3], we assessed auto-antibodies in plasma of subjects from the previous cohort [2] and the PACS subjects of the present study. Three of the PACS subjects had increased plasma levels of CHRM3-specific IgG (Fig. 2b) but not IgM or auto-antibodies directed towards β2-adrenergic receptors (data not shown).

We found that 38 of the identified DMGs were represented among the 5221 proteins that were known to be exploited by SARS-CoV-2 (Additional file 1: Fig. S2). Exploration of protein–protein interactions (PPI) to detect possible modules among these 38 DMGs revealed that most of the proteins were connected in a PPI network (Fig. 2c). PACSIN2 interacts with both the M (membrane) and N (nucleocapsid) proteins of SARS-CoV-2 and is a membrane-binding protein involved in vesicle formation. PACSIN2 also interacts with the ACE2 receptor (www.thebiogrid.org), which SARS-CoV-2 exploits for entry into cells. A regulatory module including the GTP-binding protein GNB1 (which is a central component of all five pathways listed in Fig. 2a) is linking PACSIN2 to an actin-nucleating module of ARP2/3 proteins. PIGG is an enzyme involved in membrane lipid anchor biosynthesis and interacts with 4 SARS-CoV-2 proteins, among them the M protein. The largest module in the network is composed of nucleus-encoded subunits of the mitochondrial NADH oxidase, including NDUFA10, which binds no less than 14 different SARS-CoV-2 proteins, including the E (envelope) and M proteins.

## Discussion

To the best of our knowledge, this is the first report of epigenome-wide DNAm alterations in subjects with PACS. We and others have studied DNA methylation changes in response to acute COVID-19 [4] and in healthy recoverees after the acute phase [2]. The CpG sites with the most pronounced changes comparing PACS with CC19 were found in genes encoding SNORD3B, CETP and DLGAP. SNORD3B belongs to a family of small nucleolar RNAs that have been suggested to be exploited by RNA viruses [5]. CETP is a cholesterol metabolizing enzyme and CETP-inhibitors have been proposed as treatment option for COVID-19 [6]. DLGAP is a postsynaptic protein that binds dynein (DYNLL1), which in turns binds no less than 10 SARS-CoV-2 proteins. Several of the pathways that we found epigenetically modulated in PACS subjects, including Angiotensin II receptor, muscarinic receptors and histamine signalling pathways, are relevant for the symptomatic picture of PACS. Angiotensin II has been shown to regulate the expression of ACE2 in neurons and SARS-CoV-2 binds to the ACE2 receptor via the S (spike) protein to enter cells. Histamines have been demonstrated to play a role in PACS, as partial symptom relief was reported in PACS patients upon treatment with antihistamines [7]. The pathways regulated by muscarinic receptors CHRM1&3 play a role in odour perception and were found to be epigenetically modulated in patients with PACS. Auto-antibodies towards GPCR have been demonstrated in chronic fatigue syndrome (CFS) [8] and a recent review lists a number of auto-antibodies targeting GPCRs that were identified also in PACS [3]. We found a tendency of increased levels of CHRM3-specific IgG autoantibodies in our PACS cohort. Functionally active auto-antibodies are known to disturb the balance of neuronal and vascular activity and in fact, extracorporeal apheresis could reduce levels of autoantibodies and alleviate symptoms of CFS in a recent PACS study [9]. The finding of epigenetic modulation of the pathways of the same receptors for which auto-antibodies are described in PACS and CFS is intriguing and raises the question of a possible mechanistic relationship. Hypothetically, as part of the anti-viral defense, the host could down-modulate (either by creating autoantibodies or epigenetically) pathways exploited by the virus, which when they become persistently downregulated after viral clearance, may contribute to impairment of cellular functions. In line with this idea, the PPI modules that we found to be epigenetically modulated include cellular processes that are central to vesicle formation, exemplified by the identified connection of PACSIN2 and sub-membrane actin remodeling (ARP2/3), which potentially contributes to the formation of new virus particles. Another identified module with very strong interactions with SARS-CoV-2 involves NDUFA proteins, which regulate the NADH oxidase in mitochondria. Mitochondrial dysfunction was recently described in PACS [10] and our finding of an epigenetically modified module at the core of mitochondrial function warrants further investigation in PACS. We cannot exclude the possibility that the PACS specific epigenetic signature is driven by a hidden SARS-CoV-2 reservoir. Studies including the epigenetic background and signatures in other than post viral fatigue syndromes are needed. The impact of vaccination on DNAm profiles is not possible to determine in the current study and should be evaluated in future analyses. In summary, although limited by the number of subjects included and without information on the stability of the DNAm signature, the results of the present pilot study can generate hypotheses that may help to explain the pathophysiological mechanisms underlying PACS. Hopefully, this could accelerate the development of preventive strategies and therapeutic options for PACS, which likely will persist longer than the COVID-19 pandemic itself.

## Supplementary Information

**Additional file 1**. Supplementary methods, Supplementary Figures S1–S2, Supplementary Tables S1–S3 and Supplementary References.

**Additional file 2**. Supplementary Table S4.

## Data Availability

The datasets used from Huoman J. et al. study will be available via secure token at the GeneExpression Omnibus with GEO-ID GSE178962. The data from the PACS patients analysed in the presented work will be available upon publication at the Federated European Genome-phenome Archive (FEGA) Sweden node. The datasets comprise filtered and preprocessed DNA methylation data from deidentified individual samples in the study. Utilised scripts for performing the described statistical analyses within the paper, as well as for creating graphs, will be available on the following GitHub account upon publication (https://github.com/Lerm-Lab/PACS).

## Abbreviations

ACE2: Angiotensin Converting Enzyme 2
CC19: COVID-19 Convalescent group
CETP: Cholesteryl Ester Transfer Protein
CFS: Chronic Fatigue Syndrome
CHRM3: Muscarinic acetylcholine receptor M3
COVID-19: Coronavirus Disease 2019
DMC: Differentially methylated CpG
DMG: Differentially methylated genes
EDTA: Ethylenediamine tetraacetic acid
DNAm: DNA methylation
GNB1: G Protein Subunit β 1
MDS: Multidimensional scaling
MMD: Mean methylation difference
PACS: Post-acute COVID-19 syndrome
PACSIN2: Protein Kinase C & Casein Kinase Substrate in Neurons Protein 2
PBMC: Peripheral Blood Mononuclear Cells
PCR: Polymerase chain reaction
PI3K-Akt: Phosphatidylinositol 3‑kinase-Protein kinase B
POTS: Postural orthostatic tachycardia syndrome
SARS-CoV-2: Severe Acute Respiratory Syndrome Coronavirus 2
SMIA: Suspension multiplex immunoassay
SNORD3B: Small Nucleolar RNA C/D Box 3B-1
