# Supplementary material for: Defining post-acute COVID-19 syndrome (PACS) by an epigenetic biosignature in peripheral blood mononuclear cells
Source: Clin Epigenetics. 2022 Dec 14;14:172. doi: 10.1186/s13148-022-01398-1 (PMC9748378; doi:10.1186/s13148-022-01398-1)
Supplement: Supplementary file 1 — Additional file 1. Supplementary methods, Supplementary Figures S1–S2, Supplementary Tables S1–S3 and Supplementary References. [file 13148_2022_1398_MOESM1_ESM.docx]

**Defining post-acute COVID-19 syndrome (PACS) by an epigenetic biosignature in peripheral blood mononuclear cells**

Frida Nikesjö^1^, Shumaila Sayyab^2*^, Lovisa Karlsson^2^, Eirini Apostolou^3^, Anders Rosén^3^, Kristofer Hedman^4^ and Maria Lerm^2*^

^1^Department of Respiratory Medicine in Linköping, and Department of Biomedical and Clinical Sciences, Linköping University, Linköping, Sweden

^2^Department of Biomedical and Clinical Sciences, Division of Inflammation and Infection, Linköping University, Linköping, Sweden

^3^Department of Biomedical and Clinical Sciences, Division of Cell Biology, Linköping University, Linköping, Sweden

^4^Department of Clinical Physiology, and Department of Health, Medicine and Caring Sciences, Linköping University, Linköping, Sweden

*****Corresponding authors

**ADDITIONAL FILE 1**

**incl. SUPPLEMENTARY METHODS, Supplementary Figures S1-S2, Supplementary Tables S1-S3 and Supplementary References.**

**Supplementary Table S4 (.xls) is supplied in separate Additional file 2.**

**SUPPLEMENTARY METHODS**

**Blood collection and processing of PBMC**

Blood samples in total amount of 30 ml was collected in ethylenediamine tetraacetic acid (EDTA) tubes (BD Vacutainer, no. 10331254, Fisher Scientific, Sweden). The blood samples were stored in room temperature and transported to the laboratory within 2 hours for further processing.

The tubes were kept for sedimentation in room temperature for one hour before starting the analysis. The higher plasma layer was collected in smaller tubes and centrifuged at 2000 G for 15 minutes, to receive a total amount of 6 millilitres of plasma. The plasma was saved in a freezer at -70 degrees Celsius until further analysis.

The blood tubes were pooled together and processed to isolate periferal mononuclear cells (PBMC). Isolation of PBMC from whole blood was done in SepMate tubes (SepMate^TM^, 85450, StemCell Technologies, Vancouver, Canada), prefilled with 15 millilitres of density gradient medium (Lymphoprep^TM^, no. 07811, StemCell Technologies, 06406 Bernburg, Germany). The pooled whole blood was primarily diluted with PBS containing 2% Fetal Bovine Serum (FBS) (no. 10270106, Gibco, Fischer Scientific, Sweden) before it was added to the SepMate tube, then centrifuged at 1200 G for 10 minutes to separate the layers (GE17-1440-03, GE Healthcare Life Sciences, Sigma-Aldrich, Sweden), whereafter the PBMC layer was poured into new tubes and washed in centrifugation steps (300 G for 8 minutes) to remove contaminating cells.

**DNA extraction and quantification**

DNA was extracted from the PBMC using the AllPrep® DNA/RNA Mini Kit (Cat no: 80204, Qiagen, Hilden, Germany) according to the manufacturers’ protocol. DNA concentrations were measured using the Qubit® 4.0 Fluorometer (Thermo Fisher Scientific, Waltham, Massachusetts, USA), using dsDNA High Sensitivity (HS) Assay Kit according to the manufacturers’ protocol.

**DNA methylation analysis**

The samples of extracted DNA containing 250 ng of purified DNA from PBMCs were analysed at the core facility of Bioinformatics and Expression Analysis (BEA), Karolinska Institute, Stockholm, Sweden, where the samples first went through bisulphite conversion on site, followed by the performance of the Illumina Infinium MethylationEPIC 850K array.

**Serology analysis**

Plasma was analysed for IgG antibodies against COVID-19, including anti-RBD/nucleoprotein//spike protein and nucleocapsid. The analysis was performed at the Department of Medical Microbiology at Linköping University Hospital and Department of Biomedical and Clinical Sciences, Division of Cell Biology, Linköping University, Linköping, Sweden.

Suspension multiplex immunoassay (SMIA) analysis for antibodies against CHRM3 in plasma was performed using human Muscarinic acetylcholine receptor M3 recombinant protein (#MBS955742, My Biosource).

**Descriptive analysis on demographic variables**

Initial descriptive analysis of demographic variables was performed on the available information about age, gender, smoking and BMI (kg/m2). Continuous variables were compared using an unpaired two-tailed t-test and categorical variables were examined using the Pearson χ2 test or Fisher’s exact test (if the number of observations was smaller than five).

**DNA methylation analyses**

The resulting raw IDAT-files containing the raw DNA methylation profiles for each cell type were analysed in R (v. 4.0.2) using the minfi package (v. 1.36.0) and the data were pre-processed in several steps [2]. The following filters were applied: i) removal of probes with detection p-values above 0.001, ii) removal of non-CpG probes, iii) removal of multi-hit probes, iv) removal of all probes in X and Y chromosomes. We removed the sex chromosomes from our data set, as female X-inactivation skews the distribution of β values. Of the initial 865918 probes, 813467 probes remained upon filtering with two samples removed due to low quality probes. After filtering, quality control was performed, and normalisation of the data was done with subset-quantile within array (SWAN) normalisation method [3]. The β values and M values of the samples were calculated against each probe per sample. The quality of the data was assessed before and after the normalisation. Thereafter, we performed singular value decomposition (SVD) analysis using the ChAMP package (version 2.19.3) to identify underlying components of variation within the filtered and normalised data set [4]. Significant components consisted of slide, batch and sample groups that contributed to variation within the data set. Corrections were performed for the identified components using ComBat from the SVA package (version 3.38.0) [5]. To investigate any inherent differences between three study groups (PACS, CC19 and Con), filtered, normalized and SVD corrected DNAm data were subjected to multidimensional scaling (MDS) using the Euclidean distances between the samples for 1000 most variable positions.

As PBMCs consist of multiple nucleated cell types in peripheral blood, we utilised the Houseman method to infer cell type proportions within the samples [6]. We observed differences in polymorphonuclear leucocyte proportions in individuals with PACS compared to Controls and CC19 samples (Table S2), motivating us to correcting for these cell type proportions in order to identify the differentially methylated CpGs (DMCs).

**Differential DNA methylation analysis**

As we were interested in studying CpGs that were differentially methylated between PACS and CC19s and controls from both before and after the start of the COVID-19 pandemic, we performed differential DNA methylation analysis, using the limma package (v. 3.46.0) [7]. A linear model was fitted to the filtered, normalised and SVD-corrected DNA methylation data. Identified sources of variation that were still present upon SVD correction provided the basis for the inclusion of these variables as co-variates in the models, in this case gender and neutrophil cell proportion. For each investigated probe, moderated t-statistics, log2 Fold Change (logFC) and p-values were computed. The logFC values represent the average β methylation difference (referred to as mean methylation difference, MMD) between the PACS vs CC19s and PACS vs. non-infected controls. P-values were adjusted for multiple testing using the Benjamini-Hochberg (BH) procedure for False Discovery Rate (FDR) correction. Differentially methylated CpGs (DMCs) were defined as probes having an FDR-adjusted p-value < 0.05 along with an MMD of > 0.2. The distribution of the DMCs among all investigated DNA methylation sites were illustrated by creating volcano plots (EnhancedVolcano, v. 1.8.0) [8] and heatmaps using ComplexHeatmap package in R [9]. Thereafter, the DMCs were mapped to their corresponding genes as differentially methylated genes (DMGs). DMGs contained at least one DMC and were considered hyper- or hypomethylated if all DMCs within the gene were hyper- or hypomethylated, respectively. If both hyper- and hypomethylated genes were present in the same gene, the gene was considered having a mixed methylation pattern (Additional file 2: Table S4). Furthermore, DMCs that were common between both the comparisons (PACS vs CC19s and PACS vs. non-infected controls) and with significant difference between all three groups were selected. The β values of three selected DMCs in each group were displayed in boxplots generated in R using ggplot2 package (v. 3.3.3) [11].

**Pathway analysis**

The pathway analysis was performed with the significant DMGs between PACS vs CC19 from the Panther database using the WEB-based Gene SeT AnaLysis Toolkit (WebGestalt) webserver (v. 2022) [10]. The FDR in the pathway analysis is BH adjusted p-values. We used nominal p-values (significance level set to p-value of < 0.05), in case FDR correction was too stringent. Significantly, enriched pathways were displayed in dot plots generated in R using ggplot2 package (v. 3.3.3) [11].

**DMGs Overlap to SARS-CoV-2 interactome**

In order to identify the DMGs in PACS that also interacted with the SARS-CoV-2 interactome we used the publicly available SARS-COV-2 interacting proteins and human genes curated by Biological General Repository for Interaction Datasets (BioGRID v.4.4.210, www.biogrid.org) [12]. The DMGs from PACS were compared to the SARS-CoV-2 interactome extracted from BioGRID and identified 38 overlapping genes. These overlapping genes were used as seed genes for the protein-protein interaction network from STRING-db (default confidence score)[13], which was expanded until all modules in the network were connected. The resulted module genes network was visualized in Cytoscape (v. 3.8.0) [14].

**SUPPLEMENTARY FIGURES**
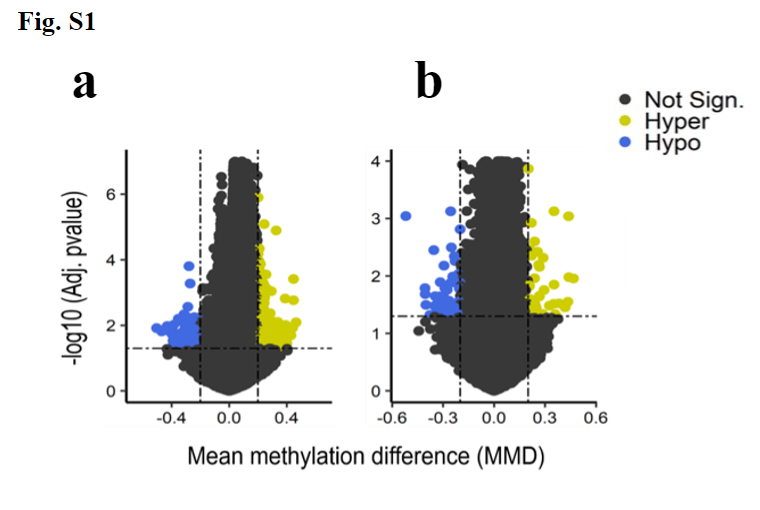


**Figure S1 – Volcano plots of differentially methylated CpG-sites in PBMC**

Enhanced volcano plot of the differentially methylated CpG-sites (DMCs) separating the PACS from the CC19 group **(a)** and PACS from the Con group **(b)**. Each dot represents a comparison of mean methylation at an individual CpG site. The x-axis with vertical line is representing a cut-off MMD > ± 0.2. The y-axis is the negative log10 of adjusted p-value with the cut off FDR p-value of 0.05 shown with dash-dotted horizontal line. Blue CpGs, hypomethylated; yellow CpGs, hypermethylated.


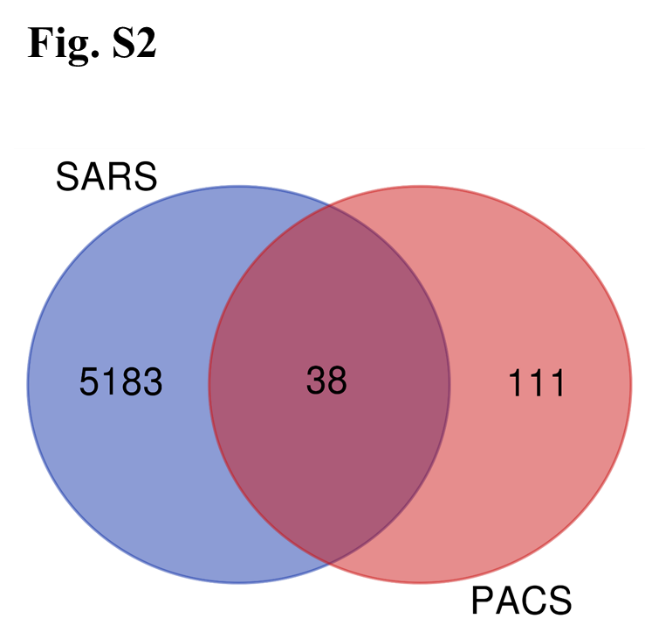


**Figure S2. PACS DMGs overlap with the SARS-CoV-2 interactome.** Venn plot showing 38 PACS specific DMGs overlaps SARS-CoV2 specific interacting proteins from BioGRID [12].

**SUPPLEMENTARY TABLES**

**Table S1a - List of PACS symptoms.** Symptoms reported by the PACS-group continuing beyond 12 weeks after the acute infection. Data from questionnaires and interviews at the inclusion date.

| **Category** | **Symptom** | **Number of subjects (n=10) (percentage)** |
| --- | --- | --- |
| Physiological | Dyspnoea | 8 (80%) |
|  | Palpitations | 7 (70%) |
|  | Muscle weakness | 6 (60%) |
|  | Dry cough | 5 (50%) |
|  | Chest discomfort/pain | 5 (50%) |
|  | Muscle/joint pain | 5 (50%) |
|  | Exercise intolerance | 3 (30%) |
|  | Throat pain | 2 (20%) |
|  | Fever | 2 (20%) |
| Neurological | Loss of smell and taste | 6 (60%) |
|  | Dizziness | 4 (40%) |
|  | Headache | 4 (40%) |
|  | Fatigue | 4 (40%) |
| Psychiatric | Anxiety/depression | 4 (40%) |
|  | Cognitive dysfunction | 3 (30%) |
|  | Insomnia/sleep disturbances | 2 (20%) |
| Other | Gastrointestinal problems | 3 (30%) |
|  | Skin problems (rash, dryness) | 3 (30%) |
|  | Eye problems (accommodation issues) | 1 (10%) |

**Table S1b - Demographic characteristics.** The table compares the included subjects with PACS with the reference material of a COVID-19 convalescent group (CC19) and controls (Con) with gender, age, BMI and smoking history. *Data is missing for five anonymous blood donors.

|  | **PACS** | **CC19** | **Con*** |
| --- | --- | --- | --- |
| Female | 7/10 (70%) | 7/14 (50%) | 13/17 (76%) |
| Age | 47.2 (27 – 57) | 44.6 (37 - 52) | 45.4 (25 - 62) |
| BMI | 25.8 (22 – 31) | 25.3 (23 – 28) | 23.4 (20 - 29) |
| Smoking history | 2/10 (20%) | 1/14 (7%) | 2/17 (12%) |

**Table S2 - Cell type proportions.** Inferred cell type proportions using the Houseman method comparing CD8 positive T-cells (CD8T), CD4 positive T-cells (CD4T), natural killer cells (NK), B-cells (B-cell), monocytes (Mono) and polymorphonuclear leucocytes (PMN).

| **Test** | **CD8T** | **CD4T** | **NK** | **B-cell** | **Mono** | **PMN** |
| --- | --- | --- | --- | --- | --- | --- |
| PACS vs Con | 0.83 | 0.38 | 0.26 | 0.54 | 0.90 | 0.01 |
| PACS vs CC19 | 0.39 | 0.48 | 0.59 | 0.16 | 0.70 | 0.01 |

**Table S3** - **Summary of methylation data.** Differentially methylated CpG sites (DMCs) and genes (DMGs) in PACS-group compared to the CC19 and Con group respectively.

| **Study group** | **PACS vs CC19** | **PACS vs Con** |
| --- | --- | --- |
| ***DMCs*** | 197 | 98 |
| Hypomethylated CpGs | 69 (35%) | 56 (57%) |
| Hypermethylated CpGs | 128 (65%) | 42 (43%) |
| ***DMGs*** | 126 | 45 |
| Hypomethylated genes | 38 (30%) | 28 (62%) |
| Hypermethylated genes | 85 (67%) | 15 (33%) |
| Mixed methylation genes | 3 (2%) | 2 (4%) |

**SUPPLEMENTARY REFERENCES**

1. Sikkeland LIB, Kongerud J, Stangeland AM, Haug T, Alexis NE. Macrophage enrichment from induced sputum. Thorax. 2007;62:558–9.

2. Aryee MJ, Jaffe AE, Corrada-Bravo H, Ladd-Acosta C, Feinberg AP, Hansen KD, et al. Minfi: a flexible and comprehensive Bioconductor package for the analysis of Infinium DNA methylation microarrays. Bioinformatics. 2014;30:1363–9.

3. Maksimovic J, Gordon L, Oshlack A. SWAN: Subset-quantile Within Array Normalization for Illumina Infinium HumanMethylation450 BeadChips. Genome Biology. 2012;13:R44.

4. Morris TJ, Butcher LM, Feber A, Teschendorff AE, Chakravarthy AR, Wojdacz TK, et al. ChAMP: 450k Chip Analysis Methylation Pipeline. Bioinformatics. 2014;30:428–30.

5. Leek JT, Johnson WE, Parker HS, Jaffe AE, Storey JD. The sva package for removing batch effects and other unwanted variation in high-throughput experiments. Bioinformatics. 2012;28:882–3.

6. Houseman EA, Accomando WP, Koestler DC, Christensen BC, Marsit CJ, Nelson HH, et al. DNA methylation arrays as surrogate measures of cell mixture distribution. BMC Bioinformatics. 2012;13:86.

7. Ritchie ME, Phipson B, Wu D, Hu Y, Law CW, Shi W, et al. limma powers differential expression analyses for RNA-sequencing and microarray studies. Nucleic Acids Res. 2015;43:e47.

8. Blighe K. EnhancedVolcano: publication-ready volcano plots with enhanced colouring and labeling [Internet]. 2022 [cited 2022 Jun 23]. Available from: https://github.com/kevinblighe/EnhancedVolcano

9. Gu Z, Eils R, Schlesner M. Complex heatmaps reveal patterns and correlations in multidimensional genomic data. Bioinformatics. 2016;32:2847–9.

10. Liao Y, Wang J, Jaehnig EJ, Shi Z, Zhang B. WebGestalt 2019: gene set analysis toolkit with revamped UIs and APIs. Nucleic Acids Research. 2019;47:W199–205.

11. ggplot2 [Internet]. [cited 2022 Jun 23]. Available from: https://link.springer.com/book/10.1007/978-0-387-98141-3

12. Stark C, Breitkreutz B-J, Reguly T, Boucher L, Breitkreutz A, Tyers M. BioGRID: a general repository for interaction datasets. Nucleic Acids Res. 2006;34:D535-539.

13. Szklarczyk D, Gable AL, Nastou KC, Lyon D, Kirsch R, Pyysalo S, et al. The STRING database in 2021: customizable protein–protein networks, and functional characterization of user-uploaded gene/measurement sets. Nucleic Acids Research. 2021;49:D605–12.

14. Otasek D, Morris JH, Bouças J, Pico AR, Demchak B. Cytoscape Automation: empowering workflow-based network analysis. Genome Biology. 2019;20:185.
